# Semantic Web integration of Cheminformatics resources with the SADI framework

**DOI:** 10.1186/1758-2946-3-16

**Published:** 2011-05-16

**Authors:** Leonid L Chepelev, Michel Dumontier

**Affiliations:** 1 Department of Biology, Carleton University, Ottawa, Canada; 2 School of Computer Science, Carleton University, Ottawa, Canada; 3 Institute of Biochemistry, Carleton University, Ottawa, Canada

## Abstract

**Background:**

The diversity and the largely independent nature of chemical research efforts over the past half century are, most likely, the major contributors to the current poor state of chemical computational resource and database interoperability. While open software for chemical format interconversion and database entry cross-linking have partially addressed database interoperability, computational resource integration is hindered by the great diversity of software interfaces, languages, access methods, and platforms, among others. This has, in turn, translated into limited reproducibility of computational experiments and the need for application-specific computational workflow construction and semi-automated enactment by human experts, especially where emerging interdisciplinary fields, such as systems chemistry, are pursued. Fortunately, the advent of the Semantic Web, and the very recent introduction of RESTful Semantic Web Services (SWS) may present an opportunity to integrate all of the existing computational and database resources in chemistry into a machine-understandable, unified system that draws on the entirety of the Semantic Web.

**Results:**

We have created a prototype framework of Semantic Automated Discovery and Integration (SADI) framework SWS that exposes the QSAR descriptor functionality of the Chemistry Development Kit. Since each of these services has formal ontology-defined input and output classes, and each service consumes and produces RDF graphs, clients can automatically reason about the services and available reference information necessary to complete a given overall computational task specified through a simple SPARQL query. We demonstrate this capability by carrying out QSAR analysis backed by a simple formal ontology to determine whether a given molecule is drug-like. Further, we discuss parameter-based control over the execution of SADI SWS. Finally, we demonstrate the value of computational resource envelopment as SADI services through service reuse and ease of integration of computational functionality into formal ontologies.

**Conclusions:**

The work we present here may trigger a major paradigm shift in the distribution of computational resources in chemistry. We conclude that envelopment of chemical computational resources as SADI SWS facilitates interdisciplinary research by enabling the definition of computational problems in terms of ontologies and formal logical statements instead of cumbersome and application-specific tasks and workflows.

## Background

The introduction and subsequent widespread availability of computers in the latter half of the 20^th ^century has had an enormous impact on chemistry and related sciences. A wide range of problems which could only be addressed by tedious manual or semi-automated computation a few decades prior suddenly became readily accessible with computers. The explosion of the diversity of the various software packages addressing every aspect of chemistry that followed can only be compared, in relative terms, to the Cambrian explosion in species diversity. Myriads of file formats, programming languages, platforms, operating systems, programming paradigms, distribution models, and access methods have been employed in hundreds of largely-independent projects, each vying for widespread adoption and often offering a unique set of functionalities and features to target a specific subdomain or application of chemistry. Consequently, computational life scientists are now obliged to spend considerable efforts on software package integration to make any progress in their daily investigations.

This problem has been especially acute for interdisciplinary studies, perhaps rising in relevance and importance with the relatively recent rise of Systems Science to prominence. For instance, to build a simple ordinary differential equation-based model of a system of partially enzyme-catalysed reactions, one may need to generate the three-dimensional structures of involved molecules, compute their energies of formation and solvation, approximate pKa values, evaluate enzyme interactions, predict kinetics, and finally solve kinetic equations, all in different software packages, which might be located on different operating systems or have unique shamanic execution procedures known only to the high priests of these packages. Even practitioners of narrower specialities are not spared the wrath of software integration, albeit on a smaller scale. Although tools to interconvert the output and input files for many of these software components have been developed [1,2], and although a number of chemical packages offer access to their functionalities through programming interfaces (e.g.[3-5]), one is left wishing that researchers in chemistry-related fields could still do more science rather than pipelining.

As scientific publishing accelerates and high-throughput experimentation platforms become increasingly pervasive, the problem of integration of the disparate computational, literature, and experimental resources is transformed from that of removing a daily nuisance to that of finding a solution without which science cannot move forward effectively. With the introduction of computational web services for life sciences (e.g. [6,7]), a step in the direction of addressing this problem has been made. With web services, tasks can be posted directly to computational resources with job execution instructions that conform to service-specific schemas, usually defined with a standard specification, most prominently the Web Service Definition Language (WSDL) [8]. Given sufficient knowledge of the service schemas, it is technically possible to automate workflow construction and provide seamless integration of web service components to fulfil a greater overarching task. In practice, however, the lack of shared and consistent schema elements with *formal semantics *has severely limited this integrative potential due to difficulties of automatically integrating service schemas themselves.

The next step of the evolution of web services was reached with the adoption of the Semantic Web and the corresponding development of Semantic Web Technologies to enable not only machine-understandable knowledge representation, but also the exposition of this knowledge and underlying concepts to automated, formal logic-based reasoning. Given a collection of knowledge triples that utilize types and relations from a formal ontology, it has finally become possible to automatically classify, integrate, and interconnect entities and concepts, much like a human expert would. To truly capitalize on this potential, simple XML-based approaches in resource specification and annotation would have to make way for Resource Description Framework (RDF) [9] and Web Ontology Language (OWL) [10] to enable automated integration of static knowledge resources with computationally generated information and provide results for cross-domain queries. This ability is indispensable in the life sciences domain to address interdisciplinary problems in toxicology or metabolism, for example. For such problems, it is not only often the case that no single database contains all the information necessary to build a working model or formulate trustworthy predictions, but it is also true that much database information is fragmented and often incomplete. Some of this missing information could be computed to fill in the gaps preventing integrative model construction, but relevant computational resources, many of which are web services, remain inaccessible to a single query method, partly due to the aforementioned integration issues. Although large collections of chemical data have recently become represented in RDF and exposed to SPARQL querying [11-13], seamless and facile integration of computational resource output to enable query completion has been difficult to attain with currently existing technologies.

Early solutions proposed for automated service integration in life sciences often drew on elements of Semantic Web Technologies. With Semantic Annotations for WSDL and XML Schema (SAWSDL), it has become possible to annotate WSDL documents with terms from formal ontologies, in a process termed 'lifting' to enable a greater degree of resource integration than with simple XML service specifications [14]. Services thus annotated would then be more readily available to integration from a central service registry. However, tight integration into the Semantic Web that would allow natural formal reasoning over such services has not yet been adequately addressed, with SAWSDL and WSDL services often requiring adaptors for service integration. The Web Service Modelling Ontology (WSMO), on the other hand, aims to construct and support a complex framework, implemented in Web Service Modelling eXecution environment (WSMX), whereby web services and all aspects of their behaviour are formally semantically represented [15].

Within the life sciences web service domain, numerous practical solutions to web service integration have been proposed, but are too numerous to completely discuss here [16]. Often, these solutions focussed on the construction of a common domain ontology, vocabulary, or registry to improve service annotation and discovery in a given domain (e.g. [17,18]). More recently, service frameworks that relied on more general ontologies for service annotation and input/output specification have also become available [19,20]. Further, frameworks integrating REST service discovery and ontology-assisted workflow composition have also appeared recently [21]. Implicit in these approaches has been the need to adopt and adhere to common service annotation, input and output specifications, or common domain ontologies. This has meant that although some of these SWS initiatives have enjoyed considerable success in enabling service interoperability and supporting facile manual workflow composition, truly seamless and automated service integration into the Semantic Web was not reached, as it was inhibited by semantic service platforms themselves.

This situation has changed with the recent introduction of the SADI framework [22]. Web services created with SADI consume and produce RDF graphs, operating on instances of input and output classes formally defined in supporting service OWL ontologies. The input class of a SADI service subsumes the output class, as these services are stateless, atomic, and annotative. That is, each service carries out a single primitive function and annotates an instance of the input class with information through a particular predicate. SADI services are also REST-like, in that there is only a standard basic set of HTTP verbs that they may respond to, namely GET and POST. A GET operation on a given service returns its semantic description, while a POST of a well-formed RDF graph to the service initiates service execution and returns the same RDF graph with the annotations created by the service. If a SADI service is computationally-intensive, standard asynchronous execution mechanisms are available.

Unlike its aforementioned predecessors, SADI service specification is extremely simple as it neither imposes nor invents a central schema, ontology, or message structure, using standard web components instead. Because of the formal logical definition of the input class, output class, and the introduced predicate, SADI services can be tightly integrated into the Semantic Web and very naturally reasoned about by a machine client. This combination of simplicity of specification and power of formal reasoning allows SADI web services to be seamlessly integrated into SPARQL queries with simple machine reasoning clients, as if the data that they can potentially generate was already available in an RDF triple store. One such prototype client, Semantic Health And Research Environment (SHARE), operates on SPARQL queries and is capable reasoning about the desired overall query goal and chaining services and information together such as to reach this goal in the least computationally expensive way [23]. SHARE draws on a central freely accessible service registry that contains information about service input, output, and predicate types to carry out this automated workflow construction. Thus, in order to pose a query through SHARE, a human agent has to be aware of service specification details in the registry to be able to create a well-formed query. For this, the user needs to acquaint themselves with the input and output classes operated upon and annotations created by the collection of SADI services available in a given SADI instance by perusing the service registry [24]. Thus, one only needs to identify an input class that contains the information that is already available as a starting point, as well as the service-specific predicates corresponding to the annotation that is desired.

In order to maximise service interoperability, it is of course recommended to reuse concepts and classes as much as possible by adhering to upper-level domain ontologies, but this is not a requirement in SADI and concepts can be manually mapped to those appearing in supporting service ontologies, if required. By the virtue of allowing external formal ontologies to be referenced in SPARQL queries executed by SHARE, this approach provides support for discourse in science while disambiguating and explicitly highlighting the points of disagreement. For example, a small molecule according to one researcher may be one that is no heavier than 500 Daltons, while another may insist that number to be 750 Daltons. With OWL, both of these viewpoints may be represented with an explicit specification which can then be used to classify a set of molecules automatically using the same SADI services, and filter down into further assertions and reasoning seamlessly. Further, multiple researchers may construct ontologies that may model molecules, and consequently *smilesmolecule *differently. So long as a formal logical mapping can be either inferred or directly made to concepts used by the service ontologies, this difference can be accommodated and the construction of computational workflows may be initiated. In other words, SADI enables liberation from conformity in existing consortia-generated ontologies and facilitates discourse and disagreement which drive science forward, at the cost of making the end user aware of the ontologies used by the existing SADI services.

**Figure 1:**
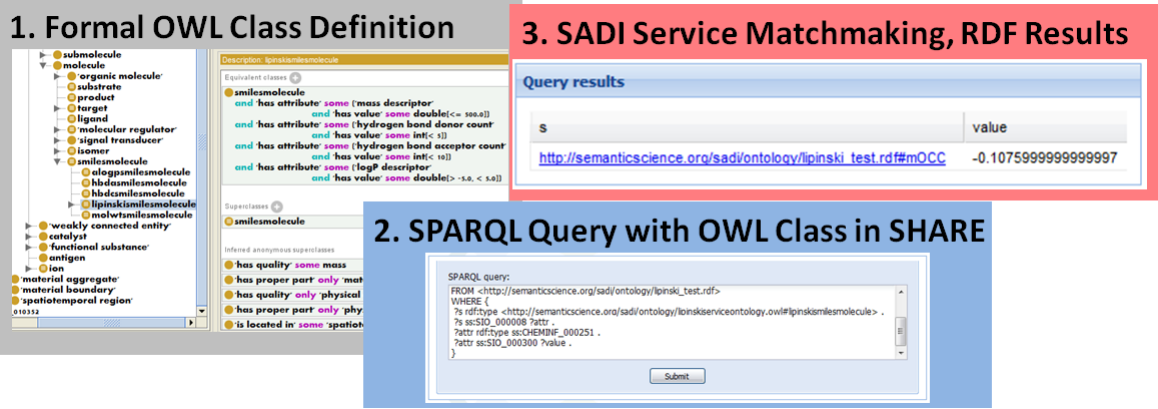
**Seamless service integration into the Semantic Web with SPARQL queries over RDF-encoded resources in chemistry, as enabled by SADI**.

SADI, with supporting machine reasoning clients, also enables the conversion of ontologies into workflows. As we have seen, a formal definition of a small molecule as having a molecular mass descriptor within a particular range in an external ontology will trigger the execution of an appropriate service through a SPARQL query posted to SHARE, if no such data is already available, and the input and output classes the service operates upon are consistent with the external ontology. Therefore, integrative workflows (in e.g. molecular classification) can be constructed just by redefining the problem in terms of classifying a given entity into a particular formal ontology-defined class or a set of classes of interest in a given study (Figure 1). Furthermore, because computational tasks are explicitly specified and service invocation is controlled, SADI allows for a greater reproducibility and interoperability of computational analysis.

In this work, we shall describe and discuss the exposition of a range of computational chemical resources as SADI services and their resultant amenability to seamless automated semantic integration through ontologies, SPARQL queries, and with graphical interfaces.

## Results and Discussion

### Exposing CDK QSAR Functionality with SADI

**Figure 2:**
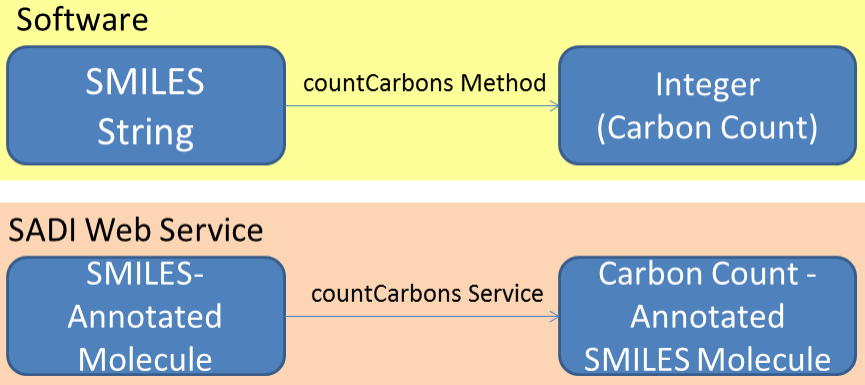
**In principle, classes and methods in APIs can, with some adjustments, be converted to input/output classes and functionality-encapsulating services**. Note that since SADI services are annotative, the input class subsumes the output class which merely contains the extra annotation computed by the service.

The exposure of computational functionality of a particular software package or application programming interface begins with the isolation of the smallest accessible functional units of the software at hand. At the most detailed level, this process may be likened to decomposition of an API into its constituent classes, which may often become input classes in the supporting service ontology, and their corresponding methods, which may be viewed as the actual computational functionality of these services (Figure 2). This comparison is limited and highly simplified, but it captures the general essence of what we are trying to do. If one concerns themselves solely with the primary *functionalities *of a given piece of software relevant to a particular problem, individual services may envelop more than one basic method in a given API or software. One limitation that arises as a consequence of the annotative nature of SADI services is the requirement to support transformative functionalities by differentiating the input and output, even if both are of the same class in the software package. For example, a method that removes hydrogen atoms from a given input molecule specified by a SMILES string and returns a SMILES string (or void), would have to be converted into a SADI service that operates upon an input class that consists of molecules that have a SMILES string specified and produces output typed to a class that consists of molecules that are annotated with a SMILES string as well as a hydrogen-free SMILES string. It must be noted that SADI services do not *have *to return all of the information present in the specification of the annotated entity that is a member of the input class. For a member of the output class of a service whose input is a molecule that has a SMILES string, it is possible to introduce only the new service-generated annotations on the existing input entity (present at a dereferenceable URI) in the output. Thus, in our hydrogen depletion example, only a reference to the input entity and the new annotation need to be included in the output, without the need to include all of the inherited entity attributes.

In this study, we have exposed a portion of QSAR descriptor calculating *functionality*, to calculate descriptors starting from a SMILES string molecular specification, as implemented in the Chemistry Development Kit. Because we are primarily concerned with demonstrating the envelopment of *functionalities *of CDK in this work, we have chosen to distribute the calculation of every available descriptor as a separate web service, even if such services relied on multiple methods and classes in CDK (Table 1).

**Table 1:** A highly abbreviated representative list of the descriptors created for this study.

Descriptor	Explanation
AlogP	Atomic calculation-based octanol-water partition coefficient.

Aromatic Atom Count	Number of aromatic atoms in a given molecule.

Atom Count	Number of atoms in a molecule.

Atomic Polarizability	Sum of atomic polarizabilities of all atoms in a given molecule.

Bond Count	Number of bonds in a molecule.

Eccentric Connectivity Index	A topological molecular descriptor that reflects on atom connectivity and distance.

Fractional Polar Surface Area	Total partially positively charged molecular surface area divided by the total molecular surface area.

Hydrogen Bond Acceptor Count	Number of atoms that can act as hydrogen bond acceptors.

Hydrogen Bond Donor Count	Number of atoms acting as hydrogen bond donors.

Ionization Potential	Propensity of a given molecule to lose an electron.

Largest Chain	The length of the longest chain of heavy atoms in a molecule

Largest PI System	The number of atoms in the largest conjugated pi-bond system.

Maximal Length/Breadth Ratio	Descriptor of molecular shape describing the ratio of a molecule's length to breadth in the region where it is highest.

Minimal Length/Breadth Ratio	Descriptor of molecular shape describing the ratio of a molecule's length to breadth in the region where it is lowest.

MlogP	Mannhold algorithm-based octanol-water partition coefficient.

Molar Refractivity	Total polarizability of a mole of a given molecule.

Molecular Complexity	A descriptor reflecting on the complexity of a given molecule in terms of quantities of heteroatoms and their connectivity.

Molecular Formula	A molecular formula that captures the types and counts of atoms present in a molecule.

Molecular Mass	The mass of a given molecule, in Daltons.

Petitjean Geometric Shape Index	A descriptor that reflects on the shape of the molecular connectivity graph and factors in distance information.

Petitjean Number	An index characterizing molecular graph topology.

Petitjean Topological Shape Index	A descriptor that reflects on the topological shape of the molecular graph.

Relative Hydrophobic Surface Area	The fraction of the overall molecular surface area that is hydrophobic.

Topological Polar Surface Area	Total molecular surface area that has a non-zero partial charge.

Total Hydrophilic Surface Area	Sum of solvent-accessible surfaces of partially charged atoms.

Total Hydrophobic Surface Area	Sum of solvent-accessible surfaces of nonpolar atoms.

Total Partially Negative Surface Area	Total surface area of a molecule that has a partially negative charge.

Total Partially Positive Surface Area	Total molecular area that has a partially positive charge.

Vertex Adjacency Magnitude	A descriptor reflecting upon the number of bonds between heavy atoms in a given molecule.

Wiener Polarity Number	A descriptor that reflects on atomic connectivity and molecular topology.

XlogP	Group contribution-based octanol-water partition coefficient.

Zagreb Index	The sum of squares of all heavy atom degrees.

A full, dynamically updated list of services is available [23].

The second step in service creation is formal description of the input and output classes in a service ontology. For our whole set of QSAR descriptor services, we have created a single service ontology [25], extended from the CHEMINF ontology [26] that contains concepts relevant to formal specification of chemical information in general and descriptor information in particular. The reuse of concepts and relations from widely accepted higher-level ontologies in CHEMINF translates into greater integration of service input and output classes into cross-domain queries.

For each descriptor calculating service, the input class is a *smilesmolecule *which is formally defined as the following.

molecule and 'has attribute' some ('SMILES descriptor' and 'has value' some string)

This input specification assures that the service will receive and operate upon an entity of the type molecule that has a SMILES descriptor and that this descriptor has a string value which the service can parse, transform into a molecular graph, and for which it can carry out descriptor calculations with a given API, in this case CDK. Note that the terms *molecule*, *has attribute*, and *has value *are reused from upper-level ontologies, meaning that concepts introduced in third-party ontologies constructed using the same upper-level ontologies will be much easier to integrate with than if we were to invent our own terms. The input is an RDF-XML graph submitted to the service through a simple HTTP POST to the service URL (Listing 1).

Listing 1. A fragment of the RDF input graph for the CDK descriptor services, in N3 form.

@prefix ss:http://semanticscience.org/.

@prefix sio:http://semanticscience.org/resource/.

ss:Ethanol rdf:type sio:SIO_011125.

ss:Ethanol sio:SIO_000008 ss:EthanolSmilesDescriptor.

ss:EthanolSmilesDescriptor rdf:type sio:CHEMINF_000018; sio:SIO_000300 "OCC".

Because the input class has to subsume the output class, the output entity has to have all the features of the input entity, but service-computed annotations should decorate the entity in the output. For instance, the definition of the output class *bondcountsmilesmolecule *for a bond count descriptor calculating service [27] is as follows.

smilesmolecule and 'has attribute' some ('bond count' and 'has value' some int)

This class definition specifies that in the output, a given *smilesmolecule *instance will be annotated with a bond count descriptor which would have an integer value (Listing 2).

Listing 2. A fragment of the RDF output graph produced by the CDK bond count calculator service, converted to N3 RDF form.

@prefix:http://semanticscience.org/sadi/ontology/lipinskiserviceontology.owl#.

@prefix ss:http://semanticscience.org/resource/.

prefix rdf:http://www.w3.org/1999/02/22-rdf-syntax-ns#.

ss:Ethanol a:bondcountsmilesmolecule,:smilesmolecule.

ss:Ethanol sio:SIO_000008 ss:EthanolSmilesDescriptor.

ss:EthanolSmilesDescriptor a sio:CHEMINF_000018; sio:SIO_000300 "OCC".

ss:Ethanol sio:SIO_000008 ss:EthanolBondCount.

ss:EthanolBondCount a sio:CHEMINF_000233.

ss:EthanolBondCount sio:SIO_000300 "3"^^http://www.w3.org/2001/XMLSchema#int.

The service OWL ontology, containing these input and output class specifications, along with the relevant predicate (*has attribute*) specifications, has to be made distributed such as to assure that these resources have dereferenceable URIs and that the ontology itself is readily available for machine reasoning agents. In order to expose services for invocation and automated workflow composition with the SHARE client, one needs to also register the service on the central SADI registry. Descriptor information can then be obtained by submitting SPARQL queries that are no different from queries over triple stores that are already populated with RDF knowledge, to the SHARE client. In essence, we are seamlessly querying *all *the data at our disposal, even the knowledge that does not yet exist, but can be generated. For example, to determine the number of hydrogen bond donors in a given molecule, one may submit the following query to the SHARE client (Listing 3).

Listing 3. A sample SPARQL query to determine the value (specified by the ?value variable) of the hydrogen bond donor count descriptor for a molecule specified in the given (lipinski_test) RDF graph, submitted to a SHARE client [28].

PREFIX rdf:http://www.w3.org/1999/02/22-rdf-syntax-ns#.

PREFIX ss:http://semanticscience.org/resource/.

PREFIX lso:http://semanticscience.org/sadi/ontology/lipinskiserviceontology.owl#.

select ?s ?value

FROMhttp://semanticscience.org/sadi/ontology/lipinskiserviceontology.owl.

FROMhttp://semanticscience.org/sadi/ontology/lipinski_test.rdf.

where {

?s rdf:type lso:smilesmolecule. #S is a molecule with a SMILES descriptor.

?s lso:hasChemicalDescriptor ?attr. #S has a chemical descriptor attr.

?attr rdf:type ss:CHEMINF_000244. #Attr is a CHEMINF H bond donor descriptor.

?attr ss:SIO_000300 ?value. #Attr must have some value.

}

This query returns the identity of the input molecule (whose SMILES descriptor is specified in the lipinski_test RDF graph), along with the value of its corresponding logP descriptor. In the background, SHARE reasons about the available services based on the information requested in the SPARQL query, *as well as the information already available in the input graph*, and automatically matches the appropriate service or services to the request. Thanks to the formal reasoning carried out by the SHARE client using the service-specific ontologies and any other ontologies referenced in the query, it is possible to infer the services needed to carry out a particular task even if the request does not use concepts identical to those found in the service definition. For example, if a predicate *hatChemischeDeskriptor *can be inferred to be equivalent to *hasChemicalDescriptor *through its formal axiomatic definition, the same set of services shall be called to fulfil queries using either predicate. Thus, the integration of SADI services into the Semantic Web by means of integration into SPARQL queries with SHARE is seamless and requires no additional programming on the part of the life science researcher.

### SADI-Enabled Format Interconversion and Software Interfacing

Though ubiquitous in chemical databases, SMILES strings do not address every chemical entity specification need. For example, one may be interested in the three-dimensional configuration of a given molecule directly, or in a more standard and canonical way of representing chemical graph structure with InChI strings [29]. Conversely, the SMILES string needed for our services to operate may not be present, but an InChI descriptor may be available instead. Finally, disparate services may operate on different formats, such that one may produce a molecule specified with an InChI string, while another may need to consume a SMILES string. Clearly, the ability to interconvert a wide range of chemical formats and representations is essential for wrapping and integrating into a single workflow the functionality of entire software packages that have no exposed programming interfaces, but are accessible for command-line interface scripting.

As a means of demonstrating the format conversion capacity as well as integration of multiple disparate software packages with SADI, we have created an Open Babel (version 2.3.0) based format conversion service to convert InChI strings to SMILES strings [30]. The implementation of this functionality and SPARQL querying for resultant data is virtually identical to that of other descriptor computing services, and is readily accessible, either through the SHARE client or through a direct POST of an RDF graph containing an instance of the *inchimolecule *class (specified below) as input.

molecule and 'has attribute' some ('InChI descriptor' and 'has value' some string)

The resultant output, by virtue of classifying into the *smilesmolecule *class, since it now contains the SMILES string representation of the queried molecule, can subsequently be consumed by all of the QSAR descriptor computing services. A collection of services to convert file formats can therefore be envisioned in order to connect multiple chemical calculation packages together, on the fly.

### Lipinski Rule of Five the Semantic Way

The simplicity of SADI architecture allows for natural computational resource integration into the Semantic Web, as demonstrated by seamless service invocation through simple SPARQL queries. The automated computational workflow construction that can be achieved thanks to this tight resource integration can be demonstrated by carrying out simple Lipinski Rule of Five analysis [31]. This well-known rule postulates that drug-like compounds can be most often characterized as having a molecular mass of less than 500 Daltons, fewer than 5 hydrogen bond donors, fewer than 10 hydrogen bond acceptors, and a logP value between -5 and 5. The definition of a Lipinski-consistent molecule lends itself quite easily for formal representation using concepts from the CHEMINF ontology, as follows.

smilesmolecule

and 'hasChemicalDescriptor' some ('mass descriptor' and 'has value' some double[< = 500.0])

and 'hasChemicalDescriptor' some ('hydrogen bond donor count' and 'has value' some int[< 5])

and 'hasChemicalDescriptor' some ('hydrogen bond acceptor count' and 'has value' some int[< 10]

and 'hasChemicalDescriptor' some ('logP descriptor' and 'has value' some double[< 5.0, > -5.0])

In this formal definition of a drug-like molecule, each statement linking the input SMILES molecule to a particular descriptor conforms to the output class and annotating predicate specification of a corresponding descriptor calculator service. This means that when a SPARQL query is posed to a SHARE client to determine whether a given instance of the *smilesmolecule *class is drug-like, the client will be capable of identifying and executing the four services necessary for the completion of this query (Listing 4).

Listing 4. A SPARQL query to determine whether a molecule (in lipinski_test RDF graph) is drug-like. If it is the case, the URI corresponding to the matching molecule will be returned.

PREFIX rdf:http://www.w3.org/1999/02/22-rdf-syntax-ns#.

PREFIX lso:http://semanticscience.org/sadi/ontology/lipinskiserviceontology.owl#.

select ?s

FROMhttp://semanticscience.org/sadi/ontology/lipinskiserviceontology.owl.

FROMhttp://semanticscience.org/sadi/ontology/lipinski_test.rdf.

where {

?s rdf:type lso:lipinskismilesmolecule.

}

The overall effect of this is that in the absence of necessary existing data, SHARE creates a web service workflow to complement the information already available, based solely on the formal definition of Lipinski drug-like molecules in a reference ontology. Not only does this lead to improvements in computational workflow reproducibility and concept disambiguation, but it also allows for straightforward means of concept reassessment from within a common framework during the course of scientific discourse. For example, the Lipinski Rule of Five has been extensively discussed, assessed and revised since its introduction [32]. By expressing their alternative definitions of drug-like compounds within the same framework as that of the original Rule of Five, it may have been possible to reduce ambiguity and inconsistencies in results stemming from inadvertent inconsistencies of data sources or precise methods of computational resource invocation. Further, the integration of service-computed data with more involved analysis (e.g. statistical regressions) as well as operations on entire data sets are possible, and demonstrations of this have been discussed at length elsewhere [22].

### Mechanisms for Parameter and Computational Experiment Provenance Specification

A number of algorithms and software packages, which may be wrapped as SADI services, require the specification of one or more parameters. The abundance of algorithm implementations and implementation-specific parameters, coupled with their under-reporting in scientific literature may often result in irreproducibility of computational experiments or discrepancies in research findings and conclusions. SADI services can specify parameters defined in an OWL ontology to control the execution of a specific computational algorithm, or to select a given algorithm from a set of equivalent algorithms which would otherwise be logically equivalent and called either together or at random, depending on the preferences and settings employed by the end user. The service description (using the GET) will display which type corresponds to the parameter class. A user wanting to specify the parameter must do so by adding its explicit description to the input RDF graph. Additionally, the provenance for the data item obtained by running the service is preserved by annotating the output as being the product of a parameterized data transformation. Besides the parameters used, this approach also allows us to explicitly specify the software (and its version), the agent (who executed it). For instance, using CHEMINF concepts, one may construct the following simplified generic output class.

molecule that

'has attribute' some ('descriptor' and 'is output of' some (

'parameterized data transformation'

and 'has agent' some 'software'

and 'has input' some 'smiles'

and 'has parameter' some (

'parameter'

and 'has value' some double

)

)

An instance of *parameterized data transformation *may be placed, along with the input, into the input RDF graph and referred to in the SPARQL query to execute service computational functionality according to explicit, precise, and reproducible specifications. To demonstrate parametric execution capacity, we have created a prototype service to compute a scaled octanol-water partition coefficient value [33]. For some compounds, it may be necessary to apply correction factors to arrive at more accurate predicted logP values. Our service computes a logP value which is multiplied by the value of the scaling factor parameter specified in the input as follows.

Listing 5. A simplified input to the parameterized logP calculating service, converted to N3.

@prefix ss:http://semanticscience.org/.

@prefix sio:http://semanticscience.org/resource/.

ss:parameterX rdf:type sio:SIO_000144; sio:SIO_000300 "1.05".

ss:Ethanol rdf:type sio:SIO_011125.

ss:Ethanol sio:SIO_000008 ss:EthanolSmilesDescriptor.

ss:EthanolSmilesDescriptor rdf:type sio:CHEMINF_000018; sio:SIO_000300 "OCC".

If no parameter is specified, the service has an internally-specified default parameter to fall back on. In both cases, the value of the parameter is reported in the output, and the parameter itself is linked to the process executed in order to obtain the value of the descriptor (Listing 6). Because the output of a parameterized service preserves this provenance information explicitly on the descriptor this service generates, it is then possible to query over only descriptors generated using a particular set of parameters, or with a given software package. This is useful when addressing the construction of toxicological models using data derived from multiple disparate data sources or across chemical entity databases. Finally, this preserved provenance information makes our calculation fully and unambiguously reproducible.

Listing 6. A simplified output of the parameterized logP calculating service, converted to N3.

@prefix:http://semanticscience.org/sadi/ontology/lipinskiserviceontology.owl#.

@prefix ss:http://semanticscience.org/resource/.

@prefix rdf:http://www.w3.org/1999/02/22-rdf-syntax-ns#.

ss:Ethanol a:bondcountsmilesmolecule,:smilesmolecule.

ss:Ethanol sio:SIO_000008 ss:EthanolSmilesDescriptor.

ss:EthanolSmilesDescriptor a sio:CHEMINF_000018; sio:SIO_000300 "OCC".

ss:Ethanol sio:SIO_000008 ss:EthanolBondCount.

ss:EthanolParamlogP a sio:CHEMINF_000251.

ss:EthanolParamlogP sio:SIO_000300 "1.727"^^http://www.w3.org/2001/XMLSchema#double.

ss:EthanolParamlogP sio:SIO_000232 ss:PDTOCCLOGP.

ss:PDTOCCLOGP:hasParameter ss:parameterX.

ss:parameterX rdf:type sio:SIO_000144.

ss:parameterX sio:SIO_000300 "1.05"^^http://www.w3.org/2001/XMLSchema#double.

### Integration and Repurposing of Chemical Resources

Service interoperability, even within a single framework, relies on the compatibility of service inputs and outputs. With SADI, formal definition of input and output classes in supporting service ontologies, especially if these ontologies draw on common upper-level concepts, facilitates service integration by enabling class equivalence inference. However, if one service produces output in terms of a molecule that has a SMILES descriptor for example, no conceivable web service framework will magically enable that output to be directly consumed by a service that demands three-dimensional molecular structure specified. In these cases, intermediary services have to be made available to bridge the gap. For example, the existing SADI service to retrieve the KEGG pathways a given drug is involved in, based on a molecule's KEGG Drug identifier [34], would have to be connected to *smilesmolecule *instance-generating services through a KEGG Drug identifier matching service.

If service input/output classes are logically equivalent or compatible, however, no such pipelining services are required to repurpose services for uses not originally anticipated. Consider, for example a functional group annotation service created by us to assist in lipid annotation and classification [35]. Given an instance of a *smilesmolecule*, this service enumerates functional group instances (from a predefined collection) occurring in the input molecule through an upper-level ontology *has proper part *predicate and produces a semantic equivalent of a chemical fingerprint in the *annotatedsmilesmolecule *output class. Although this information was originally used to classify molecules into various lipid classes, we may repurpose it for defining a customized class of chemical compounds: drug-like alkynes, as follows.

lipinskismilesmolecule and hasProperPart some Alkyl_Group

In order to invoke service execution, one needs to submit a SPARQL query, much like that for the Lipinski Rule of Five use case, to SHARE. The SHARE client will then be capable of inferring not only the necessary services to invoke in order to classify an input molecule into the *lipinskismilesmolecule *class, but would also call on the functional group annotator service to obtain *hasProperPart *annotations and complete the reasoning. Thus, it is possible to build up increasingly complex queries *ad infinitum *and let the machine reasoning clients take care of the invocation and orchestration of the web services necessary to obtain the information needed to address the query.

It is also easy to imagine a service that enumerates pharmacologically active functional groups working in conjunction with QSAR descriptor computing services to logically select compounds that are predicted to be drug-like and non-toxic, out of a large collection of combinatorially-generated chemical entities. Furthermore, thanks to the ready integration and repurposing of SADI services, it is also possible to combine QSAR descriptors with molecular pharmacological activity data to obtain a formally defined QSAR model as an output of a model creator service that could wrap existing QSAR software or mathematical scripts. Finally, it is worth stressing that due to the simplicity of SADI services, they are not precluded from working with other services, or be described and accessed through other web service frameworks.

### Exposing Chemical Database Resources as SADI Services

Web services are not solely limited to tasks involving computational capacities, but can be linked to a range of processes, including those carried out by experimental or industrial platforms. In certain cases, it is advantageous to use web services to encapsulate relational database lookup and the conversion of resultant information to RDF. Although large corpora of RDF data derived from the numerous publically accessible chemical databases have been exposed for querying through SPARQL endpoints [11,13], and although this has a proven potential in facilitating cross-domain querying in chemistry, there is a number of reasons web service-based lookups may be preferable. For example, because the major data providers do not directly publish their information in RDF there may sometimes be a delay in the conversion and incorporation of new data by the RDF triple store providers. Further, not all of the desirable information may be available in the RDF triple stores, or information might be available in a form that makes it difficult or awkward to map to one's own service ontologies, for example.

To preserve the atomic nature of SADI web services and allow for maximal flexibility in workflow construction, it is preferable to encapsulate the lookup of each index-value pair type as a separate web service in a manner identical to that of creating a CDK QSAR service. Here, the input class definition would have to require specification of the index used to look up the database and the service output class would contain entities annotated with the value or values retrieved from the database. Because this task has been demonstrated and implemented elsewhere, we shall limit our discussion of implementation to what is already stated. One point that we would like to observe is that encapsulation of database lookup functionality as SADI services allows seamless integration of chemical database resources into SPARQL queries even in the absence of corresponding RDF data. In the end, both computational and experimental resources will be available in addressing a given SPARQL query.

## Conclusions

Chemistry is indeed an immense and rapidly growing discipline with a wealth of disparate computational and database resources which are currently largely isolated and inaccessible to truly integrative queries across the entirety of the chemical (deep) web. Thus, we believe that there is an urgent need of exposing chemical resources in a manner that would be conducive to supporting a more productive way of carrying out chemical research. In this work, we have attempted to address this issue by demonstrating what we believe to be the future of chemical service distribution and chemical resource integration into the rapidly expanding Semantic Web. Using our set of SADI services to envelop the CDK QSAR-relevant descriptor functionality to decide whether a molecule was drug-like, we have demonstrated a highly integrative behaviour afforded by the simplicity of the formal semantic service specification of the SADI framework. In the future, the widespread adoption of explicit formal specification of computational tasks afforded by Semantic Web technologies may lead to an improved reproducibility and reduced ambiguity of chemical research.

With the Lipinski Rule of Five example of SHARE-assisted automated workflow construction, we have demonstrated the kinds of powerful and natural queries that could be accessible in cheminformatics research if all of the functionalities of CDK were distributed as SADI services. Although the complexity of queries amenable to SHARE automated reasoning is somewhat limited to the capacity of the supporting formal reasoning software and computational resources of the host machine, we believe that with time, this limitation shall diminish to the point of vanishing, as existing reasoners are improved and new ones become available. Engineering limitations aside, provided an ontology of common tasks and a set of adequately specified services, researchers in the future would, in principle, only need to specify their end goal or the kind of information they seek, potentially with natural language queries, and obtain it without having to be well-versed with computational tools, programming, or pipelining. At the same time, parameter-based service control would enable advanced users to express service execution specifics. Intermediate users or those wishing to specify parameters manually or string together SADI services alongside the many other cheminformatics and bioinformatics services would also be able to do this through graphical programming in the Taverna web service interface, using the Taverna SADI plugin [36]. This approach could be applied to computational queries, both big and small, because the SADI framework specifically addresses synchronous and asynchronous service execution modes. This paves the way to integration of more than just database and computational resources into scientific queries, but also potentially to automation of experimentation platforms, similar to the platform deployed for the robot scientist.

Finally, distribution of resources with SADI may act as a form of insurance against computational resources being lost into oblivion as a result of changes in platform popularity or difficulties in porting computational resources across platforms, since SADI services expose a standard, platform-independent interface. Distributing computational capacity as SADI web services in the cloud may become an attractive possibility in the future. In our future work, we intend to significantly expand our collection of web services to envelop all of chemical functionality of CDK, as well as openly accessible cheminformatics and computational packages, potentially in the cloud.

We believe that the amount of knowledge created or creatable in chemistry and related fields on a daily basis has far exceeded the potential of a single human to analyse and integrate information efficiently. In order for chemistry to progress and in order for us to handle these massive and exponentially growing amounts of data, the greater chemistry and life sciences communities have to start exploiting the power of the Semantic Web and deferring some reasoning to machine agents. We believe that the SADI web services, the semantic resource envelopment, and the seamless machine reasoning they enable constitute the first step on our journey to a way of practicing science that transcends disciplines, knows no barriers, and encompasses all human knowledge without taxing the beholder with menial and irrelevant tasks: a self-aware science.

## Methods

### Supporting Service Ontologies

We have developed a formal OWL ontology, Lipinski Service Ontology (LSO) to capture the formal definition of service input and output classes, as well as the predicate with which a given service carries out annotations. LSO is a derivative of the CHEMINF ontology for representing chemical information and chemical descriptors, and relies on an upper level ontology, Semantic Science Integrated Ontology (SIO) [37]. Within LSO, we have defined a single input class for all the services, *smilesmolecule*, and a large and growing set of output classes to correspond to the output of each service individually.

### Service Creation with CDK and OpenBabel

We implemented descriptor calculating functionality based on classes implementing the IMolecularDescriptor interface of CDK, version 1.3.0. Where a descriptor calculation returned multiple results, we created a separate service for each of the results within the descriptor vector thus returned, in order to preserve the atomic nature of SADI services. For cases where a three-dimensional molecular configuration was necessary in order to compute a particular descriptor, we employed the ModelBuilder3D class of CDK. For the InChI-to-SMILES service demonstrating the wrapping of programmatically inaccessible computational capacity distribution, we employed Java system calls to Open Babel [38] (version 2.3.0) from within the SADI service. Although we are well aware of the Open Babel API, we have chosen to access the compiled Babel binary from the command line as a means of demonstrating that numerous other command-line tools may be semantically exposed in a similar fashion.

### SHARE and SADI Service Distribution

The SHARE client and SADI skeleton for generating services are freely available for download and development. We distributed our services as Java servlets, using the Jetty servlet container. We then registered our SADI services to the central service registry and queried them on the freely accessible public SHARE interface with the queries provided in text. Functionality of SADI web services that are registered in either the central or a local service registry can also be employed in manually created workflows in Taverna through the SADI Taverna plugin.

## Authors' contributions

LLC wrote the paper and created the demonstration services. LLC and MD created the supporting service ontologies. MD contributed to the paper and provided guidance. Both authors have read and approved the final manuscript.

## Acknowledgements

This research was funded in part by NSERC CGS for LLC and the CANARIE NEP-2 Program for the C-BRASS project. We would like to thank Dr. Mark Wilkinson and Luke McCarthy for helpful discussions on SADI.

We acknowledge the article processing charge for this article that has been partially funded by Pfizer, Inc. Pfizer, Inc. has had no input into the content of the article. The article has been independently prepared by the authors and been subject to the journal's standard peer review process.
